# 654. Changing trends of molecular characteristics and antimicrobial susceptibility of hypervirulent *Klebsiella pneumoniae* in community-acquired liver abscess before and after COVID-19 outbreak in South Korea

**DOI:** 10.1093/ofid/ofad500.717

**Published:** 2023-11-27

**Authors:** Miri Hyun, Ji Yeon Lee, Hyun ah Kim

**Affiliations:** Keimyung University School of Medicine, Daegu, Taegu-jikhalsi, Republic of Korea; KEIMYUNG UNIVERSITY HOSPITAL, Daegugwangyeoksi, Taegu-jikhalsi, Republic of Korea; Department of Internal Medicine, Keimyung University Dongsan Medical Center, Daegu, Korea, Daegu, Taegu-jikhalsi, Republic of Korea

## Abstract

**Background:**

Hypervirulent *Klebsiella pneumoniae (K. pneumoniae,* hvKP*)* causes invasive infections, usually community-acquired liver abscess. In this study, we aimed to compare the molecular characteristics and antimicrobial susceptibility of hvKP in community-acquired liver abscess over times, especially before and after COVID-19 outbreak.

**Methods:**

This study was performed on *K. pneumoniae* isolates of community-acquired liver abscess collected from Keimyung University Dongsan Hospital from 2014-2015 and 2020-2021. Clinical and microbiologic data were collected from medical records. Capsular serotypes and virulence factors were identified by polymerase chain reaction using specific primers.

**Results:**

A total 79 patients were analyzed, 39 (49.4%) of 2014-2015 and 40 (50.6%) of 2020-2021. Capsular serotype K1 (61.5% vs. 62.5%) and K2 (25.6% vs. 30.0%) were no significant differences between the two periods. In virulence factors, *IutA* (82.1% vs. 97.5%) was significantly increased in 2020-2021 compared to 2014-2015 (*p*=0.028). *RmpA* (84.6% vs. 90.0%), *kfu* (61.5% vs. 70.0%), *allS* (56.4% vs. 67.5%) were showed no significant changes between two periods. Antimicrobial susceptibility of ciprofloxacin (100.0% vs. 95.0%), ceftriaxone (97.4% vs. 89.7%), and imipenem (100.0% vs. 97.5%) were no significant differences between the two periods.

**Table 1.**
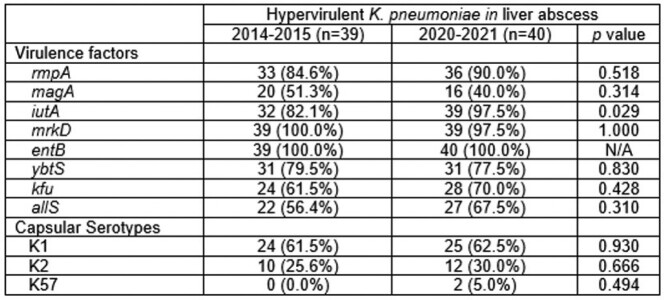
Molecular characteristics of hypervirulent Klebsiella pneumoniae in community-acquired liver abscess

**Conclusion:**

In liver abscess, the virulence factors showed a tendency to increase during the study period such as *IutA*, but the limitation is that there were small numbers of isolates included in this study. Further studies with larger number of isolates will be required to findout the changing characteristics of hvKP over times.

**Disclosures:**

**All Authors**: No reported disclosures

